# Inference of Gene Regulatory Networks with Sparse Structural Equation Models Exploiting Genetic Perturbations

**DOI:** 10.1371/journal.pcbi.1003068

**Published:** 2013-05-23

**Authors:** Xiaodong Cai, Juan Andrés Bazerque, Georgios B. Giannakis

**Affiliations:** 1 Department of Electrical and Computer Engineering, University of Miami, Coral Gables, Florida, United States of America; 2 Department of Electrical and Computer Engineering, University of Minnesota, Minneapolis, Minnesota, United States of America; University of Tokyo, Japan

## Abstract

Integrating genetic perturbations with gene expression data not only improves accuracy of regulatory network topology inference, but also enables learning of causal regulatory relations between genes. Although a number of methods have been developed to integrate both types of data, the desiderata of efficient and powerful algorithms still remains. In this paper, sparse structural equation models (SEMs) are employed to integrate both gene expression data and *cis*-expression quantitative trait loci (*cis*-eQTL), for modeling gene regulatory networks in accordance with biological evidence about genes regulating or being regulated by a small number of genes. A systematic inference method named sparsity-aware maximum likelihood (SML) is developed for SEM estimation. Using simulated directed acyclic or cyclic networks, the SML performance is compared with that of two state-of-the-art algorithms: the adaptive Lasso (AL) based scheme, and the QTL-directed dependency graph (QDG) method. Computer simulations demonstrate that the novel SML algorithm offers significantly better performance than the AL-based and QDG algorithms across all sample sizes from 100 to 1,000, in terms of detection power and false discovery rate, in all the cases tested that include acyclic or cyclic networks of 10, 30 and 300 genes. The SML method is further applied to infer a network of 39 human genes that are related to the immune function and are chosen to have a reliable eQTL per gene. The resulting network consists of 9 genes and 13 edges. Most of the edges represent interactions reasonably expected from experimental evidence, while the remaining may just indicate the emergence of new interactions. The sparse SEM and efficient SML algorithm provide an effective means of exploiting both gene expression and perturbation data to infer gene regulatory networks. An open-source computer program implementing the SML algorithm is freely available upon request.

## Author Summary

Deciphering the structure of gene regulatory networks is crucial for understanding gene functions and cellular dynamics, as well as system-level modeling of individual genes and cellular functions. Computational methods exploiting gene expression and other types of data generated from high-throughput experiments provide an efficient and low-cost means of inferring gene networks. Sparse structural equation models are employed to: i) integrate both gene expression and genetic perturbation data for inference of gene networks; and, ii) develop an efficient sparsity-aware inference algorithm. Computer simulations corroborate that the novel algorithm markedly outperforms state-of-the-art alternatives. The algorithm is further applied to infer a real human gene network unveiling possible interactions between several genes. Since gene networks can be perturbed not only by genetic variations but also by other means such as gene copy number changes, gene knockdown or controlled gene over-expression, this paper's method can be applied to a number of practical scenarios.

## Introduction

Genes in living organisms do not function in isolation, but may interact with each other and act together forming intricate networks [1]. Deciphering the structure of gene regulatory networks is crucial for understanding gene functions and cellular dynamics, as well as for system-level modeling of individual genes and cellular functions. Although physical interactions among individual genes can be experimentally deduced (e.g., by identifying transcription factors and their regulatory target genes or discovering protein-protein interactions), such experimental approach is time-consuming and labor intensive. Given the explosive number of combinations of genes involved in any possible gene interaction, such an approach may not be practically feasible to reconstruct or “reverse engineer” gene networks. On the other hand, technological advances allow for high-throughput measurement of gene expression levels to be carried out efficiently and in a cost-effective manner. These genome-wide expression data reflect the state of the underlying network in a specific condition and provide valuable information that can be fruitfully exploited to infer the network structure.

Indeed, a number of computational methods have been developed to infer gene networks from gene expression data. One class leverages a similarity measure, such as the correlation or mutual information present in pairs of genes, to construct a so-termed co-expression or relevance network [2], [3]. Another approach relies on Gaussian graphical models with edges being present (absent) if the corresponding gene pairs are conditionally dependent (respectively independent), given expression levels of all other genes [4], [5]. While the approach based on Gaussian graphical models entails undirected graphs, directed acyclic graphs (DAGs) or Bayesian networks have also been employed to infer the dependency structure among genes [6], [7]. The fourth approach employs linear regression models and associated inference methods to find the dependency among genes and to infer gene networks [8]–[11]. Finally, while these approaches use gene expression data in the steady-state, several methods exploiting time-series expression data have also been reported; see e.g., [12], [13] and references therein.

Recently, gene expression data from gene-knockout experiments have been combined with time series comprising gene expression data with perturbations to considerably improve the accuracy of network inference [14]. When a gene is knocked out or silenced, expression levels of other genes are perturbed. Different from using gene expression levels of the original network alone, comparing gene expression levels in the perturbed network with those in the original network reveals extra information about the underlying network structure. Gene perturbations can be performed with other experimental approaches such as controlled gene over-expression and treatment of cells with certain chemical compounds [8], [9]. However, these gene perturbation experiments may not be feasible for all genes or organisms. To overcome this hurdle, one can exploit naturally occurring genetic variations that can be viewed as perturbations to gene networks [15]. More importantly, such genetic variations enable inference of the causal relationship between different genes or between genes and certain phenotypes.

Several approaches are available to capitalize on both genetic variations and gene expression data for inference of gene networks. The first approach models a gene network as a Bayesian network, and then infers the network by incorporating prior information about the network obtained from expression quantitative trait loci (eQTLs) [16]–[18]. In the second approach, a likelihood test is employed to search for a casual model that “best” explains the observed gene expression and eQTL data [19]–[23]. The third approach relies on the structural equation model (SEM) to infer gene [24]–[27] or phenotype networks [28]–[34]. While these approaches focus on inference of gene networks incorporating information from eQTL, another approach employs both phenotype and QTL genotype data to jointly decipher the phenotype network and identify eQTLs that are causal for each phenotype [35]. Logsdon and Mezey [26] proposed an adaptive Lasso (AL) [36] based algorithm to infer gene networks modeled with an SEM. They compared the performance of a number of methods using simulated directed acyclic or cyclic networks. Their simulations showed that the AL-based algorithm outperformed all other methods tested. Despite its superiority over other methods, the AL-based algorithm does not fully exploit the structure of the SEM. Therefore, it is expected that a more systematic inference algorithm may significantly improve the performance of the SEM-based approach.

Motivated by the fact that gene networks or more general biochemical networks are sparse [8], [37]–[39], a sparse SEM is advocated in this paper to infer gene networks from both gene expression and eQTL data. Incorporating network sparsity constraints, a sparsity-aware maximum likelihood (SML) algorithm is developed for network topology inference. The core technique used is to maximize the likelihood function regularized by the 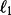-norm of the parameter vector determining the network structure. The 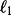-norm controls complexity of the SEM, and thus yields a sparse network. The key innovative element of the SML algorithm is a block coordinate ascent method derived to maximize the 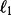-regularized likelihood function, which makes the SML algorithm computationally efficient. The simulations provided demonstrate that the novel SML algorithm offers significantly better performance than the two state-of-the-art algorithms: the AL [26], and the QDG algorithm [21]. The SML algorithm is further applied to infer a human network of 39 human genes related to the immune function.

## Results

### Sparse SEM model for gene regulatory networks

Consider expression levels of 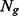 genes from 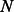 individuals measured using e.g., microarray or RNA-seq. Let 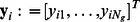 denote the 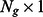 vector collecting the expression levels of these 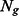 genes of individual 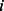. Suppose that a set of perturbations to these genes has been also observed. These perturbations can be due to naturally occurring genetic variations near or within the genes, gene copy number changes, gene knockdown by RNAi or controlled gene over-expression. In this paper, focus is placed on genetic variations observed at eQTLs, although the network model and the inference method described in the next section are also applicable to cases where other perturbations are available. As in [26], it is assumed that each gene has at least one *cis*-eQTL so that the structure of the underlying gene network is uniquely identifiable. Let 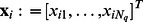 denote the genotype of 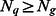 eQTLs of individual 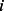. The goal is to infer the network structure of the 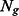 genes from the available gene expression measurements 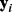, 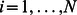, and eQTL observations 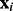, 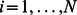.

As in [25], [26], the gene network is postulated to obey the SEM
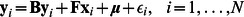
(1)where 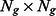 matrix 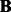 contains unknown parameters defining the network structure; 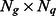 matrix 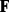 captures the effect of each eQTL; 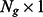 vector 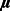 accounts for possible model bias; and 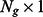 vector 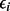 captures the residual error, which is modeled as a zero-mean Gaussian vector with covariance 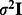, where 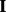 denotes the 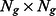 identity matrix. It is assumed that no self-loops are present per gene, which implies that the diagonal entries of 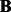 are zero. As mentioned in [26], lack of self-loops and a diagonal covariance matrix of 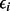 are commonly assumed in almost all graph-based network inference methods. It is further assumed that the loci of 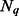 eQTLs have been determined using an existing eQTL method, but the effective size of each eQTL is unknown. Therefore, 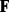 has 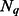 unknown entries whose locations are known and 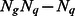 remaining zero entries (for instance 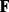 is a diagonal matrix when ).

The network inference task is to estimate 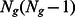 unknown entries of 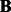, and as a byproduct, the 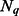 unknown entries of 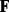. Without any knowledge about the network, no restriction is imposed on the structure specified by 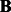. Therefore, the network is considered as a general directed graph that can possibly be a directed cyclic graph (DCG) or a DAG. Network inference is challenging since the number of unknowns to be estimated is very large for a moderately large 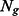. Note that under the assumption that each gene has at least one *cis*-eQTL, the “Recovery” Theorem in [26] guarantees that the network is identifiable for both DCGs and DAGs.

As discussed in [8], [37]–[39], gene regulatory networks or more general biochemical networks are sparse meaning that a gene directly regulates or is regulated by a small number of genes relative to the total number of genes in the network. Taking into account sparsity, only a relatively small number of the entries of 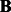 are nonzero. These nonzero entries determine the network structure and the regulatory effect of one gene on other genes. The SEM in (1) under the aforementioned sparsity assumption will be henceforth referred to as the sparse SEM. Exploiting the sparsity inherent to the network, an efficient and powerful algorithm for network inference will be developed in the ensuing section.

### Sparsity-aware inference method

Upon defining 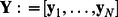, 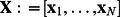, and 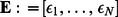, the SEM in (1) can be compactly written as 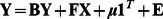, where **1** is the 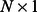 vector of all-ones. Given 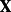 and 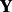, the log-likelihood function can be written as
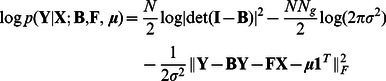
(2)where 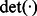 denotes matrix determinant, and 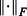 denotes the Frobenius norm.

As mentioned earlier, 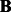 is a sparse matrix having most entries equal to zero. In order to obtain a sparse estimate of 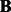, the natural approach is to maximize the log likelihood regularized by the weighed 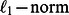 term 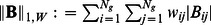, where 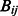 denotes the 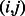
th entry of 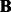. In a linear regression model, it is well known that the 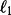-regularized least-squares estimation also known as Lasso [40] can yield a sparse estimate of the regression coefficient vector. Similarly, the 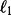-regularized maximum likelihood (ML) approach used here is expected to shrink most of the entries of 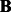 toward zero, thereby yielding a sparse matrix. It is easy to show that maximizing 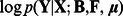 with respect to (w.r.t.) 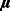 yields 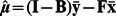, where 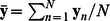 and 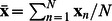. Upon defining 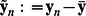, 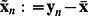, 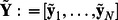, 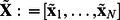, and substituting 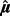 for 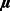 in (2), the proposed 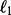-penalized ML estimation approach yields
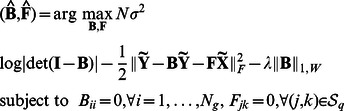
(3)where 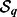 denotes the set of row and column indices of the entries of 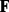 known to be zero. As assumed earlier, each phenotype has at least one *cis*-eQTL that has been identified, which implies that the locations of nonzero entries of 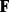 or equivalently the set 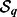 is known. However, our sparse SEM and inference method are also applicable to more general cases where some or all phenotypes have *cis*-eQTLs that have not been identified. In these cases, the locations of nonzero entries of 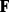 corresponding to the unidentified *cis*-eQTLs are unknown. We can form a weighted 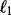-norm of the entries of 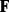 excluding those corresponding to the identified *cis*-eQTL and then add a penalty term involving this 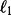-norm to the objective function in (3). This new optimization problem can be solved efficiently using a method modified from the one solving (3), as it is described in the supporting text S1.

Weights 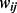 in the penalty term are introduced to improve estimation accuracy in line with the AL [36]. They are selected as 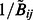, where 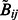 is found using a preliminary estimate of 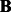 obtained via ridge regression as
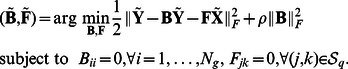
(4)The sparsity-controlling parameters 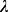 in (3) and 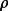 in (4) are selected via cross validation (CV), while 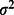 is estimated as the sample variance of the error using 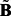 and 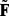. In adaptive Lasso based linear regression [36], Zou suggested using the ordinary least squares (OLS) estimate to determine the weights; if the OLS estimate does not exist due to, e.g., collinearity, Zou suggested the estimate obtained from ridge regression, although it remains to show if the ridge regression estimate is consistent in this case and if the resulting adaptive Lasso yields the desired oracle properties. If OLS is used for estimating 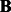 and 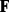 in the SEM, the solution usually does not exist since the number of unknowns is typically larger than the number of samples. However, even in this case the solution can always be obtained from ridge regression as in (4). Moreover, every entry of the solution is typically nonzero, which yields a finite weight for every variable, and thus every variable will be included in the following 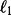-penalized ML procedure. An alternative approach is to replace the weighed 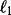-norm in (3) with an unweighted 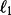-norm to obtain a preliminary estimate of 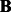 and then calculate the weights from this preliminary estimate, as in [26]. However, the unweighted 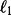-penalized ML procedure may shrink many variables to zero and exclude them from the weighted 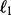-penalized ML estimator, possibly yielding a biased estimate. For this reason, the inference method in this paper uses ridge regression to determine 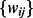, with the additional advantage of (4) admitting a closed-form solution.

**Figure 1:**
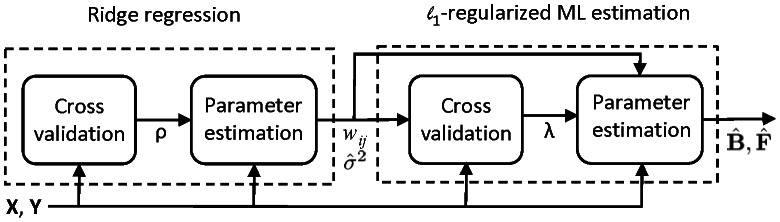
Block diagram of the sparsity-aware maximum likelihood (SML) algorithm. The first and third blocks perform cross-validation to select optimal parameters 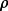 and 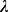 to be used in (3) and (4), respectively. The second block produces weights 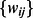 and error-variance estimate 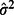 after solving (4). Finally, the fourth block takes data 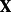 and 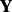 together with 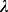, 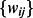 and 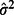 and solves (3) to yield 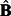, which represents the SML estimator for 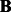 in (1) revealing the genetic-interaction network. A more detailed description of the SML algorithm is given in Algorithm 1 in the Methods section.

A block diagram of the novel inference algorithm, abbreviated as the sparsity-aware maximum likelihood (SML) algorithm, is depicted in Figure 1. The first and third blocks in Figure 1 perform cross-validation to select optimal parameters 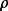 and 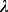 to be used in (3) and (4), respectively (see the description of the cross-validation procedure in the supporting text S1.) The third block produces weights 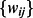 and error-variance estimate 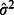 after solving (4). Finally, the fourth block takes data 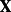 and 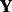 together with 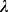, 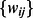 and 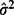 and solves (3) to yield 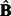, representing the SML estimator for 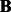 in (1) and revealing the genetic-interaction network. As it will be described in the Methods section, (4) is separable across rows of 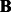 and 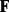, and each row of 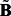 and 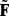 becomes available in closed form [cf. (8)–(9)]. The 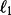-regularized ML problem (3) is solved efficiently using a novel block coordinate ascent iterative scheme given by (11)–(16) in the Methods section. Precise description of the overall SML algorithm is also presented in the Methods section as Algorithm 1, which was used to yield an executable computer program.

**Table Algorithm 1:** Algorithm 1. SML

1: Select the optimal value of 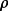 in (4), 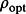 , via cross validation
2: Solve (4) with 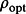 for 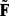 and 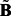
3: Estimate 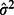 as the sample variance of E = 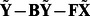
4: Compute weights 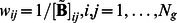
5: Compute 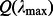 via (S2) 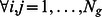
6: Compute 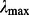 via (S9)
7: Select the optimal value of 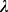 , 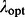 , via cross validation
8: **for** 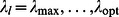 **do**
9: Compute 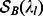 via (S4)
10: Initialize 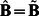 , 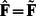 , 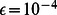 and 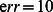
11: **while** err 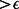 **do**
12: **for** 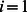 , 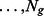 **do**
13: Obtain 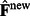 by computing its row via (7) with 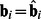
14: **end for**
15: **for** 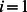 , 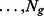 **do**
16: **for** 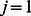 , 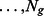 **do**
17: **if** 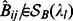 **then**
18: Compute cofactor of 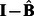 , 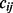
19: **if** 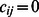 **then**
20: Compute 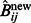 via (12)
21: **else**
22: Compute 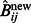 via (16)
23: **end if**
24: **end if**
25: **end for**
26: **end for**
27: Compute 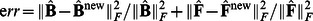
28: Set 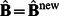 and 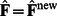
29: **end while**
30: Compute 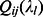 via (S1) 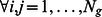
31: **end for**
32: Output 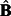 and 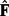 .

### Simulation studies and performance comparison of inference algorithms

In their simulation studies, Logsdon and Mezey [26] compared the performance of their AL-based algorithm with that of several other algorithms including the PC-algorithm [41], [42], the QDG algorithm [21], the QTLnet algorithm [35], and the NEO algorithm [22]. In two out of four simulation setups, the AL outperformed all other algorithms; and in the other two simulation setups, the AL and QDG algorithms exhibited comparable performance, but consistently outperformed the other two algorithms. Logsdon and Mezey [26] also considered other existing algorithms [25], [43], but these were deemed either computationally too demanding [43] or prohibitively complex [25]. For these reasons, the AL and QDG algorithms are regarded as state-of-the-art in the field. Their performance was compared against this paper's SML algorithm.

Following the setup of Logsdon and Mezey [26], two types of acyclic gene networks were simulated first: one with 10 genes and another with 30 genes. Specifically, a random DAG of 10 or 30 nodes with an expected 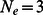 edges per node was generated by creating directed edges between two randomly picked nodes. Care was taken to avoid any cycle in the simulated graph. If an edge from node 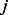 to node 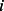 was emerging, 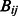 was generated from a random variable uniformly distributed over the interval 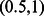 or 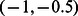; otherwise, 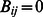. The genotype per eQTL was simulated from an F2 cross. Values 1 and 3 were assigned to two homozygous genotypes, respectively, and 2 to the heterozygous genotype. Hence, 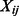 was generated as a ternary random variable taking values 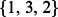 with corresponding probabilities 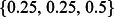. Matrix 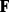 was the 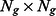 identity matrix, 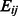 was sampled from a Gaussian distribution with zero mean and variance 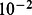, and 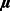 was set to zero. Finally, 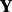 was calculated from 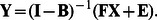


**Figure 2:**
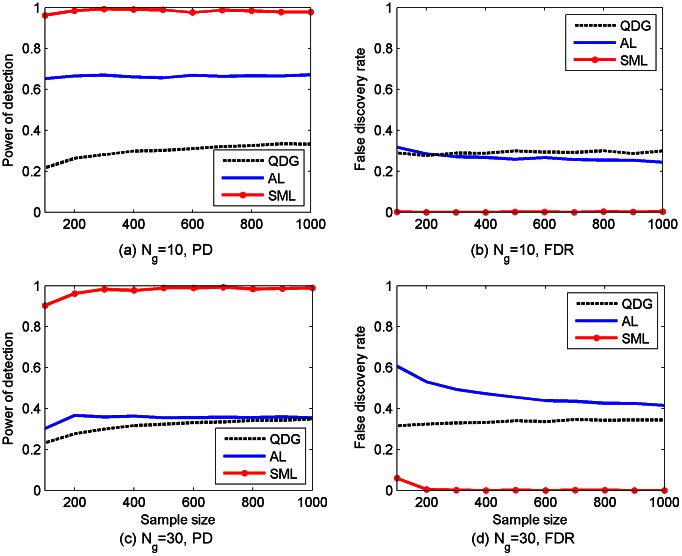
Performance of SML, AL and QDG algorithms for directed *acyclic* networks of 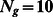 [(a) and (b)] or 30 [(c) and (d)] genes. Expected number of nodes per node is 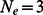. PD and FDR were obtained from 100 replicates of the network with different sample sizes (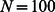 to 1,000).

For each type of gene network, 100 realizations or replicates of the network were generated, and then the SML, the AL and the QDG algorithms were run to infer the network topology. When running the SML algorithm, 10-fold CV was employed to determine the optimal values of parameters 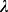 and 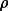 and then use these values to infer the network. An edge from gene 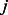 to 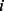 was deemed present if 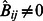. The AL algorithm also automatically ran using CV to determine the values of its parameters. For 100 replicates of the network, 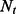 counted the total number of edges, 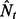 denoted the total number of edges detected by the inference algorithm. Among 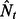 detected edges, 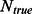 stands for the number of true edges presented in the simulated networks, and 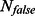 for the number of false edges. The power of detection (PD) was then found as 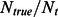, and the false discovery rate (FDR) as 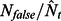. The PD and the FDR of the SML, AL, and QDG algorithms for different sample sizes are depicted in Figure 2. It is seen from Figures 2 (a) and (c) that the PD of the SML algorithm exceeds 0.9 for both networks across all sample sizes, whereas the PD of the AL algorithm is about 0.65 for 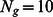 and 0.35 for 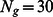. The PD of the QDG algorithm is even lower ranging from 0.22 to 0.33. As shown in Figures 2 (b) and (d), the FDR of the SML algorithm is on the order of 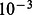 for most sample sizes, and is much lower than that of the AL and QDG algorithms, which is about 0.3 for 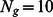 and over the range from 0.31 to 0.6 for 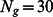.

**Figure 3:**
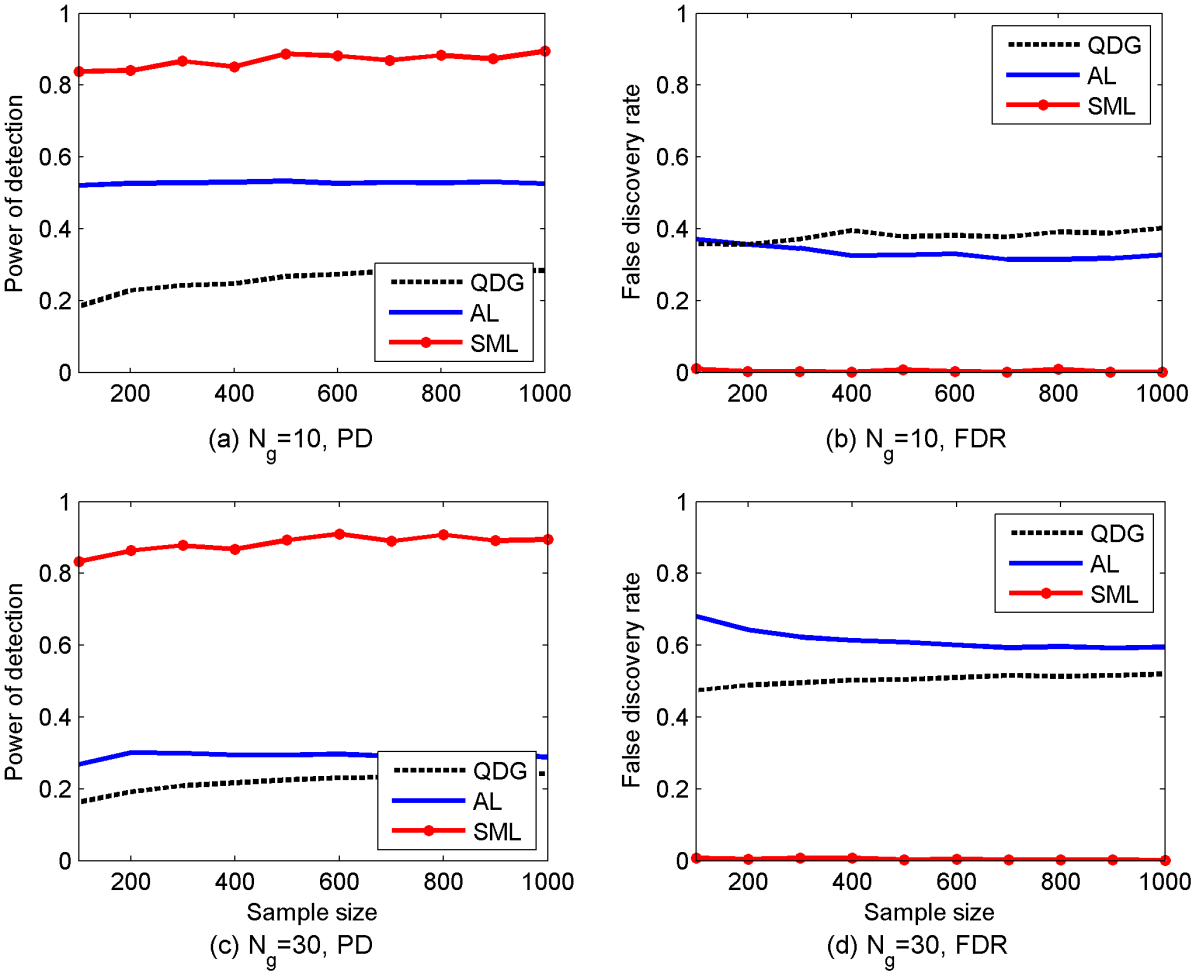
Performance of SML, AL and QDG algorithms for directed *cyclic* networks of 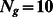 [(a) and (b)] or 30 [(c) and (d)] genes. Expected number of nodes per node is 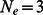. PD and FDR were obtained from 100 replicates of the network with different sample sizes (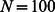 to 1,000).

Two types of *cyclic* networks were subsequently simulated: one with 10 genes and the other with 30 genes. The average number of edges per gene is again equal to 3. The same procedures used in simulating acyclic networks described earlier were employed, except that DCGs instead of DAGs were simulated. Again, 100 replicates for each type of the networks were randomly generated. The PD and the FDR of three algorithms are depicted in Figure 3. As shown in Figure 3 (a) and (c), the PD of the SML algorithm is between 0.83 and 0.9, whereas the PD of the AL algorithm is about 0.52 for 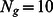 and 0.29 for 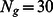, and the PD of the QDG algorithm is between 0.16 and 0.28. As shown in Figures 3 (b) and (d), the FDR of the SML algorithm is 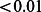, which is much smaller than that of the AL and QDG algorithms over the range from 0.33 to 0.68. For the convenience of comparison, the results in Figures 2 and 3 at sample size 500 are summarized in Table 1.

**Table 1:** Performance of SML, AL and QDG algorithms.

Network	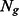	PD			FDR		
		SML	AL	QDG	SML	AL	QDG
DAG	10	0.9887	0.6564	0.3014	0.0007	0.2586	0.2991
	30	0.9891	0.3544	0.3232	0.0010	0.4548	0.3403
DCG	10	0.8872	0.5330	0.2677	0.0067	0.3268	0.3783
	30	0.8931	0.2941	0.2254	0.0020	0.6086	0.5047

Expected number of nodes per node is 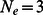. PD and FDR were obtained from 100 replicates of the network with a sample size of 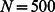.

**Figure 4:**
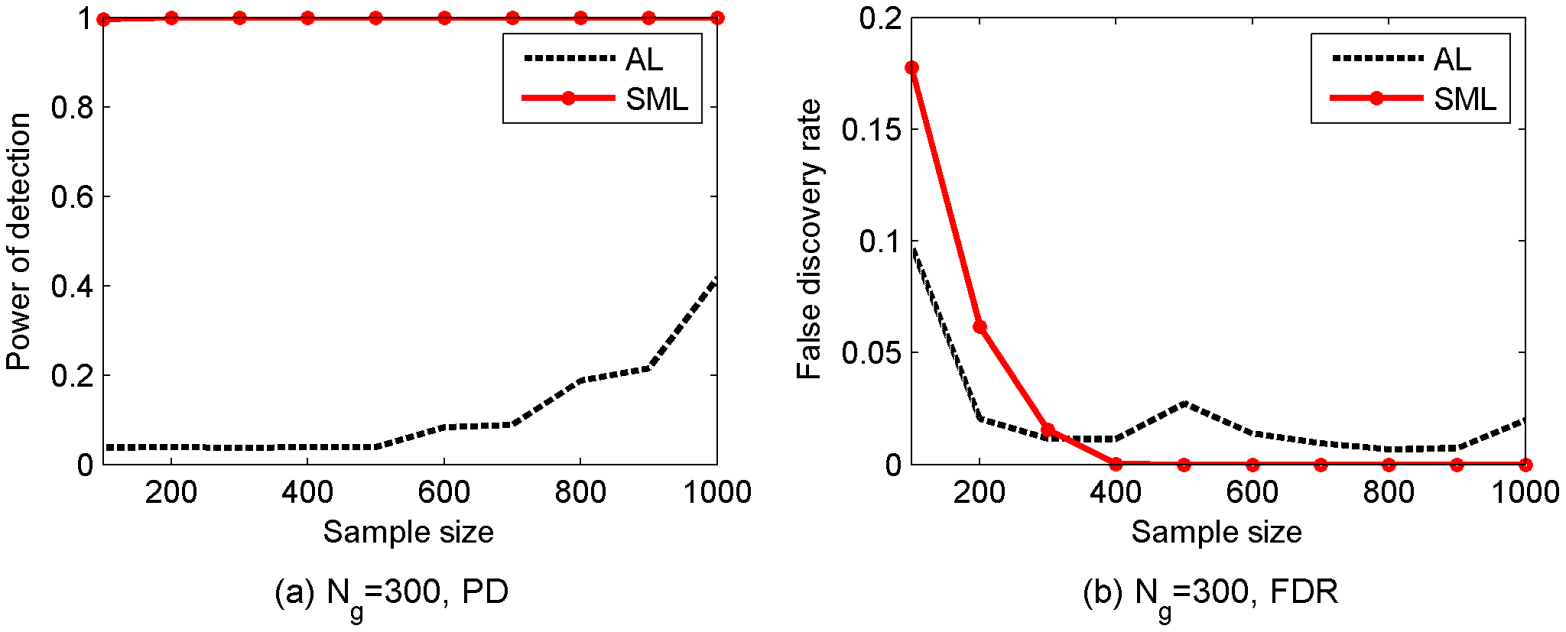
Performance of the SML and AL algorithms for directed *acyclic* networks of 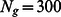 genes [(a) power of detection, and (b) false discover rate]. Expected number of nodes per node is 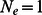. PD and FDR were obtained from 10 replicates of the network with different sample sizes (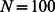 to 1,000).

As confirmed by Figures 2 and 3, the SML algorithm offers much better performance in terms of PD and FDR than the AL and QDG algorithms. However, these results were obtained for gene networks of small size. To test performance of the SML algorithm for networks of relatively large size, an acyclic network of 300 genes was simulated with an expected 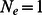 edge per node, and 10 replicates of the network were randomly generated. PD and FDR of the SML and AL algorithms obtained from these replicates are depicted in Figure 4. The PD of SML exceeds 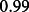 across all sample sizes from 100 to 1,000, whereas that of the AL algorithm is about 0.04 for sample sizes from 100 to 500, and gradually increases to 0.42 at the sample size of 1,000. The FDR of SML stays below 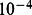 for sample sizes from 400 to 1,000, whereas the FDR of the AL algorithm is on the order of 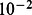 for the same sample size. When the sample size is relatively small (in the range from 100 to 300), the FDR of SML is higher than that of the AL algorithm, but it is still relatively small (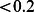). Note that the AL algorithm essentially does not work for sample sizes 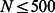, since its power is too small. All simulation results show that the novel SML algorithm significantly outperforms the AL and QDG algorithms in terms of PD and FDR.

An extra set of simulations assessing the stability of SML is described in the section of “Stability of model selection under CV with different folds” in supporting text S1, and in Figures S1 and S2. As an alternative to CV, stability selection (STS) [44] provides a means of selecting an appropriate sparsity level to guarantee that the FDR is less than a theoretical upper bound. The STS procedure was applied to the SML algorithm as described in the supporting text S1, and was used with the selection probability cutoff 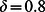 and an upper bound or target FDR = 0.1 in simulations for the networks in Figures 2[(c) and (d)] and 3 [(c) and (d)]. As shown in Figure S3, the FDR of the STS is indeed much smaller than the target FDR and almost uniform across different sample sizes, but the PD of the STS is smaller than that of CV. In fact, the FDR of the STS is on the same order as that of the CV except at the sample size of 100 for the DAG. As seen from these simulation results, although the STS guarantees a FDR upper bound, this upper bound is loose for the simulation setups tested, which may sacrifice detection power. Nevertheless, the STS procedure can select a set of stable variables as described in [44] and verified by our simulations.

**Figure 5:**
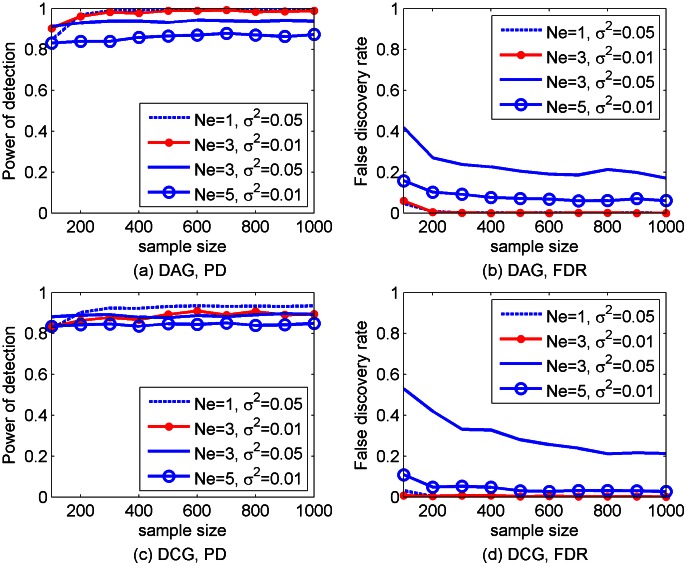
Performance of the SML algorithms for DAGs [(a) and (b)] or DCGs [(c) and (d)] of 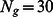 genes with an expected number of nodes per node 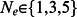 and error variance 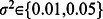. PD and FDR were obtained from 100 replicates of the network with different sample sizes (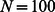 to 1,000).

So far, all the simulated data were generated with noise variance 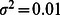. Next, the performance of SML was analyzed for simulated networks of 30 genes, when 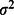 was increased to 0.05 and 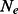 was changed from 3 to 1 or 5. Reducing 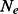 from 3 to 1 improved the performance of SML for most of the sample sizes, as it is depicted in Figure 5, withstanding the increase in the noise variance. Increasing 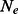 at constant 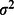, or increasing 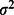 at constant 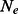 degraded the performance, most notably in the later case. Comparing Figure 5 with Figures 2 and 3 [(c) and (d)] demonstrates that in both cases the SML estimates still achieve higher detection power and lower FDR than those estimates obtained with the AL algorithm for 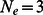 and 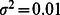.

### Inference of a network of immune-related human genes

Pickrell *et al.* [45] used RNA-Seq technology to sequence RNA from 69 lymphoblastoid cell lines derived from unrelated Nigerian individuals extensively genotyped by the International HapMap Project [46]. For each gene, they evaluated possible associations between its gene expression level calculated from RNA-Seq reads and all 3.8 million single nucleotide polymorphisms (SNPs) using the genotypes from phases II and III of the HapMap Project. At FDR = 0.1, they identified 929 genes or putative new exons that have eQTLs within 200 kb of the gene or the exon. From these 929 genes, 39 genes that are related to immune functions were selected manually by an expert as mentioned in the Acknowledgements section; expression levels and the genotypes of the eQTLs of these 39 genes in 69 individuals were used to infer the underlying regulatory network.

Pickrell *et al.* normalized expression values using quantile normalization before performing eQTL mapping. They also provided a data set that contains the number of reads mapped to each of 929 genes. This data set was obtained and the number of reads for each of 39 genes was normalized with the length of the gene to yield expression value. Such kind of values may better reflect the real expression values than the values normalized with quantile normalization, and thus they were used to infer the network. To ensure the quality of the data, the SAS ROBUSTREG procedure was applied to 69 expression values of each of 39 genes to detect outliers. The default M estimation method of the ROBUSTREG procedure was employed and the outliers were detected at a significance level of 0.05. Several gene expression values were identified as outliers since they are much larger than the remaining values that were classified as non-outliers. The outliers were replaced with the largest non-outlier. More sophisticated means of revealing and imputing outliers are possible using robust statistical schemes; see e.g., [47]. The genotypes of the eQTLs of the 39 genes were downloaded from HapMap database using the SNP IDs for the eQTL provided by Pickrell *et al.*. About 12% genotypes are missing. These missing genotypes were imputed using the program IMPUTE2 [48]. The name and a brief description of each gene were obtained from DAVID [49] using the Ensembl gene IDs provided by Pickrell *et al.* Information of these 39 genes including their Ensembl gene IDs and names, a brief description of each gene, and HapMap SNP IDs of the associated eQTLs can be found in Table S1 in the supporting information.

**Figure 6:**
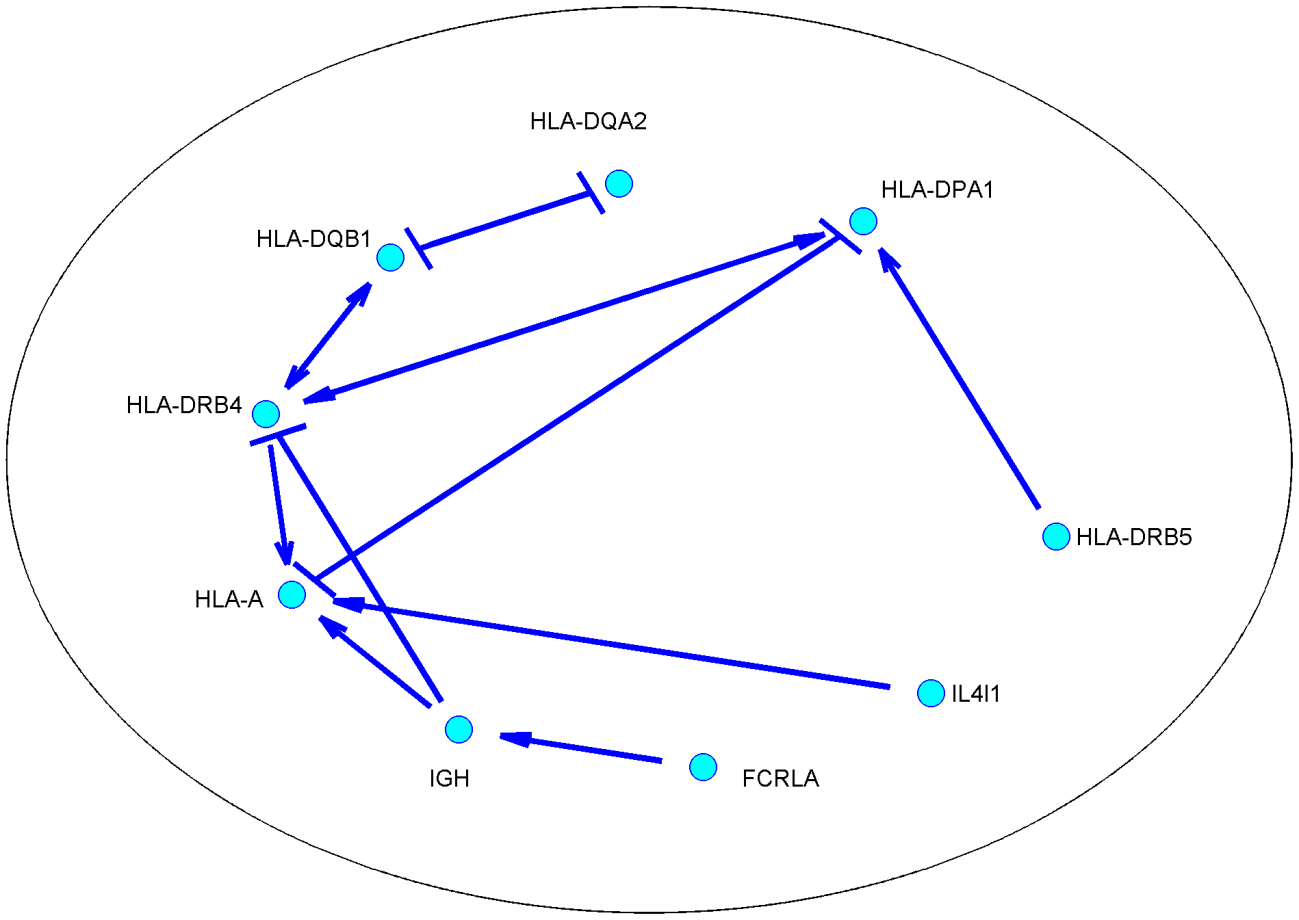
The network of 39 human genes inferred from gene expression and eQTL data with the SML algorithm. The 39 genes related to the immune function were chosen from [45] to have a reliable eQTL per gene. The SML algorithm was run with stability selection and edges were detected at an 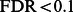. See Table 3 for the IDs and description of 39 genes. IGH in this figure corresponds to gene ID ENSG00000211897. A 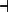 edge stands for inhibitory effect and a 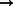 edge stands for activating effect.

The SML algorithm was run with the expression levels and genotypes of eQTLs of these 39 genes. An edge from gene 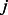 to 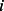 was detected if 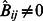. To improve the reliability of the detected edges, the SML algorithm was run with stability selection at an FDR 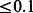 using 100 random subsamples, yielding 13 directional edges as shown in Figure 6. The frequency of each edge detected in 100 runs is given in Table S2. It is interesting to see from Figure 6 that only 9 genes are involved in the network, and the remaining 30 genes are not connected with any other genes and thus not shown in the figure. AL and QDG algorithms were also run with stability selection at an FDR 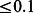 using 100 random subsamples. The edges detected by AL and QDG algorithms and their frequencies are included in Table 4. The AL algorithm detected only one edge that was not detected by the SML algorithm. The QDG yielded 3 edges, one of which was also detected by the SML algorithm. The relatively small number of edges detected by three algorithms was likely due to relatively low signal-to-noise ratio (SNR) in this data set. The estimated noise variance was 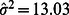 and the estimated SNR was 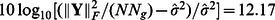 dB, which was much lower than that (about 25.8 dB) in the case of 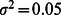 in Figure 5. However, comparing the results of three algorithms shows that our SML algorithm detected more edges than the other two algorithms at the same FDR due to its higher detection power as confirmed also by the simulations. When the FDR was increased to 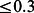, the SML algorithm with stability selection yielded a network of 16 genes that have 42 edges as shown in Figure S4 in the supporting information. Since only 39 genes were used to construct the network, an edge between two genes may not necessarily imply a direct regulatory effect, but may reflect the fact that two genes are either directly linked or very close to each other in the real network that consists of all genes. Particularly, if two genes are co-regulated by another gene which is not included in the 39 genes, these two genes may have a unidirectional or bidirectional edge.

Most edges in Figure 6 are between major histocompatibility complex (MHC) genes (HLA-A, HLA-DPA1, HLA-DQA2, HLA-DQB1, HLA-DRB4 and HLA-DRB5), which is expected since these genes may interact with each other and/or be co-regulated. FCRLA is a member of Fc receptor-like family of genes. It is expressed in B cells and interacts with IgG and IgM [50], [51]. IGH, encoding the heavy chain of immunoglobulin, characterizes the B-cell origin of the samples. Hence, it is not surprising to see an edge between FCRLA and IGH. Interleukin-4-induced gene 1 (IL4I1) was first described in the mouse [52] and subsequently characterized in human B cells [53]. Human IL4I1 is expressed by antigen-presenting cells [54], which may allude to the edge between HLA-A and IL4I1, but this may be speculative since there is no edges between IL4I1 and MHC class II genes in the network. The edges between IGH and HLA-A and between IGH and HLA-DRB4 may reflect the coordinated effect of antibody and MHC as a response to antigens. In fact, IGH is connected to most of MCH genes in Figure S4, which may imply the wide coordination between the two classes of molecules.

## Discussion

Integrating genetic perturbations with gene expression data for inference of gene networks not only improves inference accuracy, but also enables learning of causal regulatory relations among genes. Although much progress has been made recently on the development of inference methods that integrate both types of data, a truly efficient algorithm is missing. The SEM provides a systematic framework to integrate both types of data, and offers flexibility to model both directed cyclic as well as acyclic graphs. However, there is no systematically designed inference method for SEMs of relatively high dimension, which is particularly true for gene networks typically including hundreds or thousands of genes. Traditionally, inference for SEMs has relied on the ML or generalized least-squares methods implemented with a numerical optimization algorithm [55], [56]; but recently, Bayesian alternatives [57] have emerged too, based on Markov chain Monte Carlo simulations [58], [59]. These methods not only are computationally intensive, but also may be inaccurate for sparse SEMs of relatively high dimension, since they do not account for sparsity present in the model.

In the context of QTL mapping, Newton's method is employed in [27] to implement the ML method, while the genetic algorithm [60], [61] is used in [24], [25] to maximize the likelihood function, and in conjunction with a model selection method using a 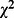 test or Occam's window to search for the best network topology. These methods are not scalable to SEMs of relatively high dimension. The AL-based algorithm proposed in [26] is more efficient because it automatically incorporates variable selection into the inference process, and also takes into account the sparsity present in gene networks. However, the AL-based scheme borrows the adaptive Lasso [36] optimally designed for the linear regression model instead of the SEM. In contrast, the SML algorithm proposed in this paper directly maximizes the 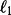-regularized likelihood function of the SEM, which fully exploits the information present in the data and therefore improves inference accuracy. Moreover, the novel block coordinate ascent method combined with discarding rules can efficiently maximize the 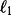-regularized likelihood function, rendering the SML algorithm applicable to SEMs of high dimension. However, unlike the AL-based algorithm, the SML algorithm maximizes a non-convex objective function as given in (3). Although the “Recovery” Theorem in [26] guarantees the identifiability of the network, the algorithm can converge to a local maximum that may not necessarily be coincident with the global maximum corresponding to the optimal network. A common technique for alleviating this problem is to use multiple random initial values. We tested multiple initial values in our simulations and observed that the algorithm converged to the same solution. In Algorithm 2, we used the pathwise coordinate optimization strategy as used in [62], where the solution of (3) obtained with 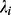 was used as the initial point for the run with 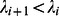. The pertinence of this strategy is corroborated by simulated numerical tests, showing significant performance gains of the SML algorithm in terms of detection power and FDR when compared to the AL-based algorithm.

Comparisons in the Simulation Studies section, as summarized in Figures 2–5, demonstrated that the SML algorithm markedly outperforms two state-of-the-art algorithms: the AL [26] and QDG [21] algorithms. For three directed acyclic networks with number of genes 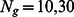 and 300, respectively, the PD of the SML algorithm exceeds 0.9 for all sample sizes from 100 to 1,000, and is greater than 0.99 for most sample sizes. This is much greater than the PD of the AL and QDG algorithm that ranges from 0.004 to 0.67. In fact, The QDG algorithm was too time-consuming to obtain results for 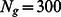. The FDR of SML is on the order of 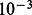 for most sample sizes, which is much smaller than those of the AL and QDG algorithms, that are between 0.25 and 0.6 for 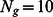 and 30. The FDR of the AL algorithm for 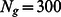 is between 0.02 and 0.1. The only case where the FDR of SML exceeds that of the AL algorithm is when 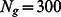, and the sample size 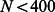. However, the AL algorithm essentially does not work in this case, since its PD is about 0.04. In the case of directed cyclic networks, all algorithms offer slightly degraded performance when compared to that of directed acyclic networks. However, the SML algorithm still considerably outperforms the AL and QDG algorithms.

Using a limited amount of available data [45], 39 genes related to the immune system and having one eQTL per gene were selected to infer a possible network among these genes. At an FDR 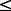
10% for the detected edges, a network of 9 out of 39 genes containing 13 edges were obtained. An edge between two genes in the inferred network may be an indication of the direct regulator effect, or indirect interaction or co-regulation mediated by some other genes that are not among the 39 genes. The majority of the edges were reasonably expected from the experimental results in the literature, while the remaining edges may represent new interactions to be elucidated.

Structural equation modeling has a long history of about a century, with well-documented contributions to various fields including biology, psychology, econometrics and other social sciences [55], [56], [63], [64]. The model considered in this paper belongs to a class of SEMs with observed variables [55]. The SML algorithm is the first one that is systematically developed for inferring sparse SEMs with observed variables. It is expected to accelerate the application of high-dimensional SEMs not only in biology, but also in other fields.

## Methods

### Ridge regression

#### Closed-form solution

Problem (4) can be solved row by row independently in closed form. Let 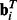, 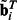, 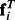, 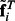 and 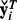 denote the 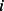
th row of 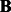, 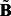, 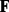, 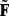, and 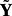, respectively. Then, problem (4) is equivalent to the following problem
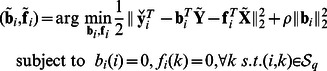
(5)where 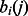 stands for the 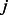
th element of 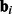 and 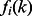 denotes the 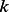
th element of 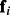.

The constraints in (5) can be imposed directly by discarding elements of 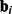 and 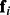 known to be zero. To this end, define an 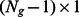 vector 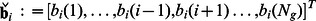 and a vector 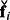 collecting the entries of 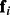 whose indexes are not in 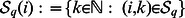. Let 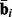 and 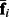 denote the solution for 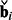 and 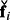, respectively. Similarly, let 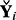 be a sub-matrix of 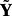 formed by removing the 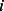
th row of 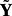, and 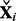 collecting those rows of 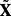 whose indexes are not in 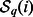. Under these definitions, (5) is equivalent to
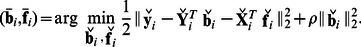
(6)


Minimizing for 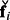 first, one arrives at
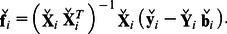
(7)Substituting (7) into (6) after defining 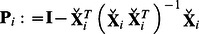, yields
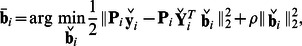
which is a standard ridge regression problem with solution given by
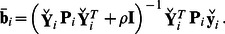
(8)Finally, substituting (8) into (7) yields
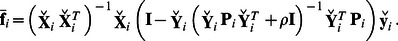
(9)Vectors 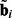 and 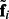 are obtained by inserting zeros into 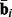 and 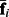 at appropriate positions specified by the constraints in (5). Collecting 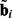 and 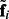, 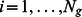, yields the solution of (4), namely 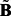 and 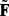.

Parameter 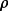 is required to solve (4). A 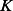-fold CV scheme is adopted for this purpose with typical choices of 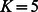 or 10, as suggested in [65]. A detailed description of the CV procedure [65] is given in supporting text S1.

### 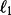-regularized ML method

#### Coordinate-ascent algorithm

Solving (3) is performed by a cyclic block-coordinate ascent iteration. Consider a specific cycle where estimates of 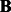 and 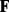 obtained in the previous cycle are denoted by 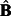 and 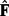, respectively. The first step of the cycle entails maximizing the objective function in (3) w.r.t. 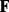 with 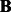 fixed to 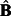, which yields a new estimate of 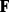 denoted as 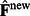. This step coincides with the minimization of the objective function in (4) w.r.t. 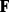, which admits a closed-form solution per row given by (7). In each of the next 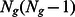 steps of the cycle, the objective function in (3) is maximized w.r.t. a single entry of 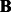, namely 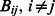, with the remaining entries of 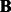 equal to the corresponding entries of 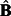 and 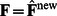. An expression for the new estimate of 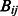, 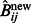 is derived next.

Define matrix 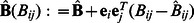 having all entries equal to those of 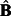 except for its 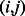
th entry, which is replaced by the variable 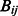, where 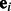 and 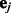 denote the 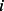
th and 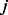
th canonical vectors in 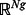, respectively. Then, the objective in (3) can be written as
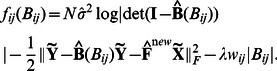
(10)Upon re-arranging and discarding constant terms, (10) simplifies to
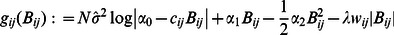
(11)where 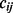 denotes the 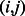
th co-factor of matrix 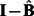, and 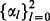 are defined as
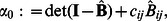


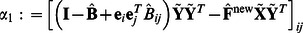


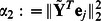
with 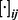 representing the 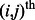 entry of the matrix between brackets. For numerical stability and computational savings, all co-factors 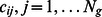, per row can be computed simultaneously by solving 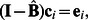 with 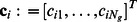. After an iteration step is completed and 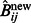 is computed, 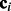 can be updated using the matrix inversion lemma as 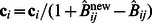 before updating 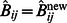.

A new estimate of 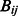 is formed by maximizing 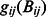 in (11). To this end, consider two cases with 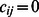 and 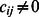. If 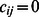, the logarithmic term can be dropped from (11) yielding a standard Lasso problem with solution
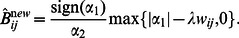
(12)When 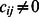, three hypotheses are tested, namely: i) 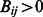; ii) 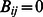; and, iii) 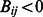. For hypotheses i) and iii), the solution can be found in closed form after equating to zero the derivative of (11) w.r.t. 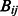. The roots found in both cases have to be tested against the corresponding hypothesis. Then, the surviving roots are grouped with 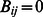 as candidate solutions, and the candidate yielding the maximum 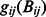 is the new estimate 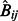.

Specifically, under hypothesis i) where 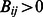, the derivative of 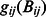 in (11) takes the form 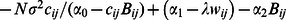, which upon multiplication with 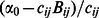 turns into
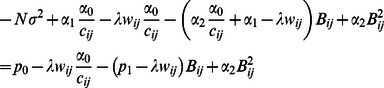
(13)under the definitions
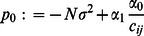


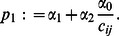
Consider the equation obtained by setting (13) equal to zero. If it has root(s), then they are given by
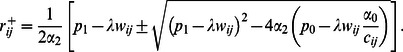
(14)Let 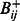 stand for the set containing the positive root(s) in (14). If the equation does not have a solution, 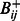 equals the empty set.

Similarly for hypothesis iii) where 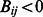, setting the derivative of (11) equal to zero, one obtains an equation. If this equation has root(s), they are given by
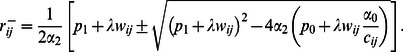
(15)Let 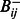 denote the set containing the negative root(s) in (15). If the equation does not have a solution, 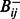 becomes the empty set. Considering all three hypotheses, one arrives at
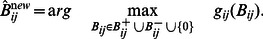
(16)After a cycle is completed, the algorithm is checked for convergence by verifying whether the inequality 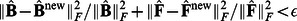 is satisfied, where 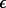 is a prespecified small constant. If yes, the algorithm is stopped and 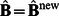 and 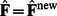 are output as the final estimates of 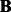 and 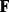; otherwise, 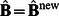 and 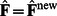 and one proceeds to execute the next cycle.

In order to increase the speed of the SML algorithm, the discarding rules proposed for sparse linear regression [66], [67] were adapted to the sparse SEM setup. Given 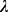, the discarding rules provide a means of computing a matrix 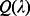, whose entries determining entries of 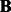 that can be set to zero *a priori* without be updated during the coordinate-ascent iterations. A detailed description of the discarding rules, together with the CV procedure to select the optimal 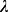, and the expression for the required 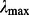, that is, the minimum value of 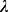 for which the solution to (3) is null, are provided in the supporting text S1.

### SML algorithm

The overall SML approach described in the Methods section, including the ridge regression weights, the discarding rules, and the coordinate descent cycle is depicted step-by-step in Algorithm 1. The for-loop starting from line 8 and ending at the last line is the 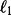-regularized ML method for computing 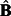 and 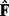 in (3), which comprises the block coordinate ascent algorithm and discarding rules. In our computer program, these lines were written as a subroutine. Since the CV on line 7 needs to solve (3), the subroutine is also called on line 3 with 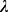 varying from 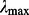 to 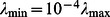. An additional subroutine implementing ridge regression was written to solve (4), and subsequently called on lines 1 and 2.

In the supporting text S1, three relevant extensions to the SML algorithm are described. First, stability selection [44] is applied to the SML, as an alternative to CV, to select the sparsity level so that the FDR is controlled. Second, the SML is extended to handle heteroscedasticity in the SEM error. Third, the SML is modified to enable inference of unknown eQTLs. In addition, supporting text S1 gives a description of the state-of-the-art AL-based and QDG algorithms that were considered for comparison with SML.

## Supporting Information

Text S1 **Supporting text.**
(PDF)

Figure S1
**Performance of the SML algorithm for DAGs [(a) and (b)] or DCGs [(c) and (d)] of** 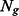 **= 30 genes obtained with 5 (solid line) or 10 (dashed line) fold cross validation.** Expected number of nodes per node is 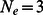. PD and FDR were obtained from 100 replicates of the network with different sample sizes from 100 to 1,000.(TIF)

Figure S2
**Performance of the SML algorithm for DAGs [ (a) and (b)] or DCGs [(c) and (d)] of** 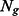 **= 30 genes obtained with the optimal** 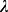 **(solid line) or an** 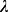 **10% less than the optimal** 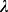 **(dashed line).** Expected number of nodes per node is 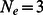. PD and FDR were obtained from 100 replicates of the network with different sample sizes from 100 to 1,000.(TIF)

Figure S3
**Performance of the SML algorithm with stability selection (STS) or cross validation for DAGs [ (a) and (b)] or DCGs [(c) and (d)] of** 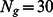 **genes.** Expected number of nodes per node is 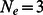. PD and FDR were obtained from 100 replicates of the network with different sample sizes from 100 to 1,000.(TIF)

Figure S4
**The network of 39 human genes inferred from gene expression and eQTL data with the SML algorithm.** The 39 genes related to the immune system were chosen from [45] to have a reliable eQTL per gene. The SML algorithm was run with stability selection and edges were detected at an 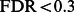. See Table S1 for the IDs and description of 39 genes. IGH in this figure corresponds to gene ID ENSG00000211897. A 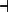 edge stands for inhibitory effect and a 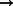 edge stands for activating effect.(TIF)

Table S1 **Thirty nine immune-related human genes used to infer a network.**
(XLSX)

Table S2 **Edges of the gene network in** **Figure 6** **inferred with the SML algorithm and edges detected with AL and QDG algorithms.**
(XLSX)

Software S1 **Software package implementing the SML algorithm.**
(ZIP)
