# Supplementary material for: Inference of Gene Regulatory Networks with Sparse Structural Equation Models Exploiting Genetic Perturbations
Source: PLoS Comput Biol. 2013 May 23;9(5):e1003068. doi: 10.1371/journal.pcbi.1003068 (PMC3662697; doi:10.1371/journal.pcbi.1003068)
Supplement: Text S1 — Supporting text. (PDF) [file pcbi.1003068.s001.pdf]

# Inference of gene regulatory networks with sparse structural equation models exploiting genetic perturbations
# Supporting text S1

Xiaodong Cai[1,*], Juan Andrés Bazerque[2], Georgios B. Giannakis[2]
**1 Department of Electrical and Computer Engineering, University of Miami, Coral Gables, FL 33146, USA**
**2 Department of Electrical and Computer Engineering, University of Minnesota, Minneapolis, MN 55455, USA**
**∗ E-mail: x.cai@miami.edu**

# Cross-validation

## Cross-validation for ridge regression

The solution of (4) requires specifying the parameter $\rho$. A $K-$fold cross-validation (CV) scheme is adopted for this purpose with typical choices of $K = 5$ or 10, as suggested in [1]. For $\kappa = 1, \ldots, K$ the dataset is divided in two parts, namely $(\tilde{\mathbf{Y}}_\kappa, \tilde{\mathbf{X}}_\kappa)$ with $N_s/K$ samples and $(\tilde{\mathbf{Y}}_{(-\kappa)}, \tilde{\mathbf{X}}_{(-\kappa)})$ with the remaining $(K-1)N_s/K$ samples. For each value of $\rho$ on a grid of $R = 30$ points regularly spaced in logarithmic scale between $10^{-6}$ and 1, the solution to (4) computed using $(\tilde{\mathbf{Y}}_{(-\kappa)}, \tilde{\mathbf{X}}_{(-\kappa)})$ is denoted as $(\tilde{\mathbf{B}}_{(\rho,\kappa)}, \tilde{\mathbf{F}}_{(\rho,\kappa)})$. The error $e_\kappa(\rho) := \|\tilde{\mathbf{Y}}_\kappa - \tilde{\mathbf{B}}_{(\rho,\kappa)}\tilde{\mathbf{Y}}_\kappa - \tilde{\mathbf{F}}_{(\rho,\kappa)}\tilde{\mathbf{X}}_\kappa\|_F^2$ is obtained and averaged across folds to obtain the error estimate $e(\rho)$. The value of $\rho$ that attains a minimum $e(\rho)$ is selected as the optimal value. In order to save computations, the grid of $\rho_r$ values is scanned progressively for $r = 1, \ldots, R$. The procedure is stopped when $e(\rho_{r-1}) < e(\rho_r)$, and $\rho_{r-1}$ is chosen as the optimal value.

## Cross-validation for $\ell_1$-regularized ML estimation

The CV procedure for selecting $\lambda$ follows the steps used to select $\rho$ in ridge regression. The sample is divided into $K$ folds, and for $\kappa = 1, \ldots, K$ the $\kappa$-th fold is set aside for validation. For $L$ values of $\lambda$ between $\lambda_{\min} = 10^{-4}\lambda_{\max}$ and $\lambda_{\max}$, the solution to (3) computed using $(\mathbf{Y}_{(-\kappa)}, \mathbf{X}_{(-\kappa)})$ is denoted as $(\hat{\mathbf{B}}(\lambda,\kappa), \hat{\mathbf{F}}(\lambda,\kappa))$, and the validation error is computed for each $\kappa$ using $(\hat{\mathbf{B}}(\lambda,\kappa), \hat{\mathbf{F}}(\lambda,\kappa))$ and $(\mathbf{Y}_\kappa, \mathbf{X}_\kappa)$. Upon averaging the validation errors across $\kappa$, an optimal $\lambda$ is selected as the largest parameter that minimizes this mean-CV error within one standard deviation.

## Stability of model selection under CV with different folds

A set of simulations were run to test robustness of the SML algorithm. First, the fold number of CV was changed from $k = 5$ to $k = 10$ for the DAGs of 30 genes in Figures 2(c) and 2(d) and the DCGs of 30 genes in Figures 3(c) and 3(d) with an expected number of edges $N_e = 3$. As shown in Figure S1, $k = 5$ and $k = 10$ offer almost identical performance. Simulations with a suboptimal $\lambda$ that is 10% less than the optimal $\lambda$ obtained from 5-fold CV were then run for the networks used in Figure S1. As expected, the suboptimal $\lambda$ yielded slightly worse performance as shown in Figure S2; the performance degradation is very small for the DAGs but relatively large for the DCGs, which implies that it is important to choose the optimal value of $\lambda$.

# Discarding rules

In Lasso regression, it is known that for a given $\lambda$ some predictors can be set to zero *a priori* without solving the Lasso inference problem [2,3]. Hence, these predictors can be discarded when inferring other

predictors. Rules for discarding predictors were also derived in [2,3]. In particular, the strong rules in [3] can discard a large number of predictors, which significantly reduces the computation needed to solve the Lasso problem. To reduce computational burden and improve the speed of the SML algorithm, the technique in [3] is employed next to derive strong rules for setting some entries of matrix $\mathbf{B}$ to zero *a priori*, before running the coordinate-ascent algorithm.

Let $\hat{\mathbf{B}}(\lambda)$ and $\hat{\mathbf{F}}(\lambda)$ denote the optima of (3) for a given $\lambda$. Let also $Q_{ij}(\lambda)$ stand for the derivative of the differentiable part of (3); i.e., $N\sigma^2 \log|\det(\mathbf{I}-\mathbf{B})| - \frac{1}{2}\|\tilde{\mathbf{Y}} - \mathbf{B}\tilde{\mathbf{Y}} - \mathbf{F}\tilde{\mathbf{X}}\|_F^2$, w.r.t. $B_{ij}$ evaluated at $\hat{\mathbf{B}}(\lambda)$ and $\hat{\mathbf{F}}(\lambda)$. Then, $Q_{ij}(\lambda)$ can be found as

$$Q_{ij}(\lambda) = -\frac{N\sigma^2 c_{ij}(\lambda)}{\det(\mathbf{I}-\hat{\mathbf{B}}(\lambda))} + \left[\tilde{\mathbf{Y}}\tilde{\mathbf{Y}}^T - \hat{\mathbf{B}}(\lambda)\tilde{\mathbf{Y}}\tilde{\mathbf{Y}}^T - \hat{\mathbf{F}}(\lambda)\tilde{\mathbf{X}}\tilde{\mathbf{Y}}^T\right]_{ij} \tag{S1}$$

where $c_{ij}(\lambda)$ is the $(i,j)$th co-factor of $\mathbf{I}-\hat{\mathbf{B}}(\lambda)$, and $\sigma^2$ can be estimated as $\hat{\sigma}^2 = \frac{1}{NN_g}\|\tilde{\mathbf{Y}} - \hat{\mathbf{B}}(\lambda)\tilde{\mathbf{Y}} - \hat{\mathbf{F}}(\lambda)\tilde{\mathbf{X}}\|_F^2$. Let $\lambda_{\max}$ denote the smallest value of $\lambda$ that yields $\hat{B}_{ij}=0$, $\forall i,j$ (an expression for $\lambda_{\max}$ will be given later). After recognizing that $c_{ij}(\lambda_{\max}) = 1$, it follows that

$$Q_{ij}(\lambda_{\max}) = -N\sigma^2 + \left[\tilde{\mathbf{Y}}\tilde{\mathbf{Y}}^T - \hat{\mathbf{F}}(\lambda_{\max})\tilde{\mathbf{X}}\tilde{\mathbf{Y}}^T\right]_{ij} \tag{S2}$$

where $\hat{\mathbf{F}}(\lambda_{\max})$ is obtained by substituting $\mathbf{B}=\mathbf{0}$ into (7). Note that $Q_{ij}(\lambda_{\max})$ can be computed without knowledge of $\lambda_{\max}$.

For $\lambda < \lambda_{\max}$, the discarding rule is given by

$$|Q_{ij}(\lambda_{\max})| < w_{ij}(2\lambda - \lambda_{\max}) \;\Rightarrow\; \hat{B}_{ij}(\lambda) = 0. \tag{S3}$$

When trying to find solutions of (3) along a path of $\lambda$ defined with a decreasing set of values $\lambda_0 = \lambda_{\max} > \lambda_1 > \ldots > \lambda_{\min}$, which are needed in CV, the following alternative rule is possible:

$$|Q_{ij}(\lambda_{l-1})| < w_{ij}(2\lambda_l - \lambda_{l-1}) \;\Rightarrow \hat{B}_{ij}(\lambda_l) = 0. \tag{S4}$$

Let $\mathcal{S}_B(\lambda_l)$ denote the set of $\hat{B}_{ij}(\lambda_l) = 0$ obtained from (S4) or (S3).

The rationale behind rules (S3) and (S4) is described in the following. By the optimality of $\hat{\mathbf{B}}(\lambda)$, the KKT condition implies that

$$Q_{ij}(\lambda) = \lambda w_{ij} s_{ij} \tag{S5}$$

where $s_{ij}$ is the subgradient of $|B_{ij}(\lambda)|$, and $s_{ij}=1$ if $\hat{B}_{ij}(\lambda) > 0$, $s_{ij}=-1$ if $\hat{B}_{ij}(\lambda) < 0$, or, $s_{ij} \in [-1,1]$ if $\hat{B}_{ij}(\lambda) = 0$. Taking the derivative w.r.t. $\lambda$ on both sides of (S5) results in $\frac{dQ_{ij}(\lambda)}{d\lambda} = \left(s_{ij} + \lambda\frac{ds_{ij}}{d\lambda}\right) w_{ij}$. Thus, under the assumption that $\left|s_{ij} + \lambda\frac{ds_{ij}}{d\lambda}\right| \leq 1$ (see [3] for a discussion on this assumption), it follows that

$$\left|\frac{dQ}{d\lambda}\right| \leq w_{ij}. \tag{S6}$$

Applying the mean-value theorem between $\lambda_l$ and $\lambda_{l-1}$ yields

$$|Q(\lambda_{l-1}) - Q(\lambda_l)| \leq w_{ij}(\lambda_{l-1} - \lambda_l). \tag{S7}$$

If the inequality in (S4) holds, then (S7) implies $|Q(\lambda_l)| < \lambda_l w_{ij}$, which in accordance with (S5) yields $|s_{ij}| < 1$ and thus $\hat{B}_{ij}(\lambda_l) = 0$, as specified by rule (S4). Similarly, one can justify rule (S3).

### Computation of $\lambda_{\max}$

When $\lambda$ is sufficiently large such that $\hat{\mathbf{B}} = \mathbf{0}$, (S5) and the definition of $s_{ij}$ imply that

$$\left| \frac{Q_{ij}(\lambda)}{w_{ij}} \right| \leq \lambda, \ \forall i, j = 1, \ldots, N_g. \tag{S8}$$

Since $Q_{ij}(\lambda) = Q_{ij}(\lambda_{\max})$ for $\lambda > \lambda_{\max}$ as indicated in (S2), we obtain

$$\lambda_{\max} = \max_{i,j=1,\ldots,N_g} \left| \frac{Q_{ij}(\lambda_{\max})}{w_{ij}} \right|, \tag{S9}$$

being the minimum possible value satisfying (S8) and thereby giving rise to a $\lambda$ yielding $\hat{\mathbf{B}} = \mathbf{0}$ in (3). Substituting $Q_{ij}(\lambda_{\max})$ from (S2) into (S9) yields $\lambda_{\max}$. Recall from (S2) that $Q_{ij}(\lambda_{\max})$ can be computed without knowledge of $\lambda_{\max}$.

## Extensions of the SML algorithm

### Stability selection

In Algorithm 1, CV is used to select the optimal value of $\lambda$ that determines the level of sparsity in $\hat{\mathbf{B}}$. However, it was observed that a single run of CV may not yield a consistent estimate of variables [4,5]. An alternative approach to choosing appropriate variables is stability selection (STS) [6] that offers a theoretical upper bound on the FDR. We next describe the procedure for applying STS to our SML algorithm. Upon drawing $N_{STS}$ random data subsamples of size $N_s = \lfloor N/2 \rfloor$, where $\lfloor N/2 \rfloor$ stands for the largest integer less than $N/2$, (3) is solved per subsample and per $\lambda$, yielding a collection of estimates $\hat{\mathbf{B}}_\nu(\lambda)$, $\nu = 1, \ldots N_{STS}$, $\lambda = \lambda_{\min}, \ldots, \lambda_{\max}$. Defining an $N_g \times N_g$ matrix $\mathbf{T}(\lambda) := \sum_{\nu=1}^{N_{STS}} \text{abs}(\text{sgn}(\hat{\mathbf{B}}_\nu(\lambda))$ whose $(i,j)$th entry counts the nonzero $[\hat{B}_\nu(\lambda)]_{ij}$'s across $\nu = 1, \ldots, N_{STS}$ estimates, edge $(i,j)$ is declared as stably identified at level $\lambda$, if $T_{i,j}(\lambda)$ exceeds a threshold $\delta N_{STS}$ with $\delta \in (0.6, 0.9)$. For a given $\lambda$, an upper bound on the FDR resulting from the STS procedure is given by $\overline{\text{FDR}}(\lambda) := \frac{q^2}{(2\pi-1)N_g^2 q_s}$ [6], where $q$ denotes the average number of nonzeros in $\hat{\mathbf{B}}_\nu(\lambda)$ across $\nu = 1, \cdots, N_{STS}$ estimates, and $q_s$ the average number of stably identified edges. Both $q$ and $q_s$, and thus $\overline{\text{FDR}}(\lambda)$, increase as the sparsity-controlling parameter $\lambda$ decreases. Therefore, the optimal $\lambda$ denoted as $\lambda_{STS}$ for a target $\overline{\text{FDR}}$ is selected as the smallest $\lambda$ satisfying $\overline{\text{FDR}}(\lambda) \leq \overline{\text{FDR}}$. The overall result presents low sensitivity on frequency $\delta$, since a higher and more restrictive $\pi$ is automatically compensated for by a lower more permissive $\lambda$. Note that the original STS procedure [6] employs the random LASSO where the weights are randomly selected from some specified values. In our case, we do not use random weights but still use the weights obtained from ridge regression, since our simulations show that those weights yield improved performance.

### Heteroscedasticity

Removing the assumption that the residual error $\boldsymbol{\epsilon}_i$ in (1) has covariance matrix $\sigma^2\mathbf{I}$, the SML algorithm can be extended to the more general case where the covariance of $\boldsymbol{\epsilon}_i$ is a diagonal matrix $\mathbf{R} = \text{diag}(\sigma_1^2, \cdots, \sigma_{N_g}^2)$ with unequal diagonal entries $\sigma_i^2, i = 1, \cdots, N_g$. In this case, the log-likelihood function in (2) becomes $\log p(\mathbf{Y}|\mathbf{X}; \mathbf{B}, \mathbf{F}, \boldsymbol{\mu}) = \frac{N}{2} \log|\det(\mathbf{I}-\mathbf{B})|^2 - \frac{N}{2} \log[\det(R)] - \frac{NN_g}{2} \log(2\pi) - \frac{1}{2}\text{Tr}[(\mathbf{Y} - \mathbf{BY} - \mathbf{FX} - \boldsymbol{\mu}\mathbf{1}^T)^T \mathbf{R}^{-1} (\mathbf{Y} - \mathbf{BY} - \mathbf{FX} - \boldsymbol{\mu}\mathbf{1}^T)$, where $\text{Tr}(\cdot)$ denotes the trace of the matrix in parentheses. It is easy to show that maximizing this likelihood function w.r.t. $\boldsymbol{\mu}$ yields the same expression for $\boldsymbol{\mu}$ as the one obtained earlier by maximizing the likelihood function in (2). Then the objective function in ridge regression problem (4) becomes $J_{\text{ridge}} = \frac{1}{2}\text{Tr}[(\tilde{\mathbf{Y}} - \mathbf{B}\tilde{\mathbf{Y}} - \mathbf{F}\tilde{\mathbf{X}})^T \mathbf{R}^{-1} (\tilde{\mathbf{Y}} - \mathbf{B}\tilde{\mathbf{Y}} - \mathbf{F}\tilde{\mathbf{X}})] + \rho\|\mathbf{B}\|_F^2 =$

$\sum_{i=1}^{N_g}\left[\frac{1}{2\sigma_i^2}\|\check{\mathbf{y}}_i^T - \mathbf{b}_i^T\tilde{\mathbf{Y}} - \mathbf{f}_i^T\tilde{\mathbf{X}}\|_2^2 + \rho\|\mathbf{b}_i\|_2^2\right]$. Therefore, it is again possible to solve (4) row by row separately, but replace the objective function in (5) with $\sum_{i=1}^{N_g}\left[\frac{1}{2}\|\check{\mathbf{y}}_i^T - \mathbf{b}_i^T\tilde{\mathbf{Y}} - \mathbf{f}_i^T\tilde{\mathbf{X}}\|_2^2 + \rho_i\|\mathbf{b}_i\|_2^2\right]$, where $\rho_i = \rho\sigma_i^2$. Specifically, problem (5) can be solved with this new objective function and a specific value $\rho_i$ that is obtained from CV performed separately for each row. Variance $\sigma_i^2$ is then estimated as the residual error for the $i$th row obtained with estimated $\mathbf{b}_i$ and $\mathbf{f}_i$. The $\ell_1$-regularized ML problem (3) can also be reformulated by replacing the objective function with the following one: $J_{\mathrm{ML}} = N\log|\det(\mathbf{I}-\mathbf{B})| - \frac{1}{2}\mathrm{Tr}\big[(\tilde{\mathbf{Y}} - \mathbf{B}\tilde{\mathbf{Y}} - \mathbf{F}\tilde{\mathbf{X}})^T\mathbf{R}^{-1}(\tilde{\mathbf{Y}} - \mathbf{B}\tilde{\mathbf{Y}} - \mathbf{F}\tilde{\mathbf{X}})\big] - \lambda\|\mathbf{B}\|_{1,W}$. With this new objective function, (11) becomes $g_{ij}(B_{ij}) = N\hat{\sigma}_i^2\log|\alpha_0 - c_{ij}B_{ij}| + \alpha_1 B_{ij} - \frac{1}{2}\alpha_2 B_{ij}^2 - \lambda\hat{\sigma}_i^2 w_{ij}|B_{ij}|$, where $\hat{\sigma}_i^2$ is the estimate of $\sigma_i^2$. Therefore, the coordinate-ascent algorithm can be easily modified by replacing $\hat{\sigma}^2$ with $\hat{\sigma}_i^2$ and $w_{ij}$ with $w_{ij}\hat{\sigma}_i^2$ in $g_{ij}(B_{ij})$ to estimate $B_{ij}$.

### Identification of eQTLs

The SML algorithm can be extended to handle the case where some or all phenotypes have unidentified *cis*-eQTLs, if a new penalty term, that involves the weighed $\ell_1$-norm of the entries of $\mathbf{F}$ excluding those corresponding to the identified *cis*-eQTL, is added to the objective function in (3). In this case, it is only necessary to modify line 13 of the SML algorithm as follows. Consider redefining $\check{\mathbf{f}}_i$ as the one that contains the entries of $\mathbf{f}_i$ corresponding to the known *cis*-eQTLs and let $\check{\mathbf{f}}_i'$ contain the remaining entries of $\mathbf{f}_i$. Similarly, let $\check{\mathbf{X}}_i$ collect rows of $\tilde{\mathbf{X}}$ corresponding to the known *cis*-eQTLs and $\check{\mathbf{X}}_i'$ contain the remaining rows of $\tilde{\mathbf{X}}$. Then on line 13 of the SML algorithm, (7) is replaced by $\check{\mathbf{f}}_i = \left(\check{\mathbf{X}}_i\check{\mathbf{X}}_i^T\right)^{-1}\check{\mathbf{X}}_i\left(\check{\mathbf{y}}_i - \check{\mathbf{Y}}_i\check{\mathbf{b}}_i - \check{\mathbf{X}}_i'^T\check{\mathbf{f}}_i'\right)$ with $\mathbf{f}_i'$ taking values obtained in the previous iteration. The entries of $\mathbf{f}_i'$ can be updated using the coordinate ascent method in the glmnet algorithm [7] for Lasso based linear regression.

## State-of-the-art algorithms

### Adaptive Lasso-based algorithm

The AL-based algorithm [8] involves three basic steps: the first one performs standard eQTL mapping to identify a *cis*-eQTL per gene; the second one applies the adaptive Lasso [9] to infer the SEM; and the third step performs a permutation test to ensure that edges in the network obtained from the second step correspond to correct dependencies in the directed graph. Since the core of the AL-based algorithm is the adaptive Lasso in step 2, it is described here briefly for completeness. The adaptive lasso estimates $\mathbf{B}$ and $\mathbf{F}$ as follows

$$(\hat{\mathbf{B}},\hat{\mathbf{F}}) = \arg\max_{\mathbf{B},\mathbf{F}} -\frac{1}{2}\|\tilde{\mathbf{Y}} - \mathbf{B}\tilde{\mathbf{Y}} - \mathbf{F}\tilde{\mathbf{X}}\|_F^2 - \lambda\psi_W(\mathbf{B},\mathbf{F})\} \tag{S10}$$
$$\text{subject to } \ B_{ii} = 0, \forall i = 1,\ldots,N_g, \ \ F_{jk} = 0, \ \forall(j,k)\in\mathcal{S}_q$$

where $\psi_W(\mathbf{B},\mathbf{F}) := \sum_{i=1}^{N_g}\sum_{j=1}^{N_g} w_{ij}|B_{ij}| + \sum_{i=1}^{N_g}\sum_{j=1}^{N_q} v_{ij}|F_{ij}|$. Weights $w_{ij}$ and $v_{ij}$ are given by $w_{ij} := |\tilde{B}_{ij}|^{-1/2}$ and $v_{ij} := |\tilde{F}_{ij}|^{-1/2}$, where $\tilde{B}_{ij}$ and $\tilde{F}_{ij}$ are obtained by solving the following Lasso problem

$$(\tilde{\mathbf{B}},\tilde{\mathbf{F}}) = \arg\max_{\mathbf{B},\mathbf{F}} -\frac{1}{2}\|\tilde{\mathbf{Y}} - \mathbf{B}\tilde{\mathbf{Y}} - \mathbf{F}\tilde{\mathbf{X}}\|_F^2 - \rho\psi(\mathbf{B},\mathbf{F})\} \tag{S11}$$
$$\text{subject to } \ B_{ii} = 0, \forall i = 1,\ldots,N_g, \ \ F_{jk} = 0, \forall(j,k)\in\mathcal{S}_q$$

with $\psi(\mathbf{B},\mathbf{F}) := \sum_{i=1}^{N_g}\sum_{j=1}^{N_g}|B_{ij}| + \sum_{i=1}^{N_g}\sum_{j=1}^{N_q}|F_{ij}|$. Constants $\lambda$ and $\rho$ are obtained via CV. We obtained the program implementing the AL-based algorithm from the authors of [8] and used it in our simulation studies. In this program, the glmnet algorithm [7] is employed to solve Lasso problems (26) and (27).

### QDG Algorithm

The QDG algorithm [10] first builds an undirected graph for the phenotypes under consideration, using an undirected dependency graph [11] or a skeleton derived from the PC algorithm [12]. It then orients edges in the undirected graph by using a score calculated from the likelihood of the data for different edge directions. The edge orientation process is performed iteratively for each edge until no edge changes its direction. We obtained the program implementing the QDG algorithm from the authors [10] and used the default settings of the program in our simulations.

## References

1. Hastie T, Tibshirani R, Friedman J (2009) The Elements of Statistical Learning: Data Mining, Inference, and Prediction. New York: Springer, 2 edition.

2. El Ghaoui L, Viallon V, Rabbani T (2010) Safe feature elimination in sparse supervised learning. Technical Report UC/EECS-2010-126, EECS Dept., University of California at Berkeley.

3. Tibshirani R, Bien J, Friedman J, Hastie T, Simon N, et al. (2012) Strong rules for discarding predictors in lasso-type problems. J R Statist Soc B 74: 245266.

4. Hall P, Lee ER, Park BU (2009) Bootstrap-based penalty choice for the Lasso, achieving oracle performance. Statistica Sinica 19: 449-471.

5. Martinez J, Carroll R, Müller S, Sampson J, Chatterjee N (2011) Empirical performance of cross-validation with oracle methods in a genomics context. The American Statistician 65: 223–228.

6. Meinshausen N, Bhlmann P (2010) Stability selection. J R Statist Soc B 72: 417–473.

7. Friedman J, Hastie T, Tibshirani R (2010) Regularization paths for generalized linear models via coordinate descent. J Stat Softw 33: 1-22.

8. Logsdon BA, Mezey J (2010) Gene expression network reconstruction by convex feature selection when incorporating genetic perturbations. PLoS Comput Biol 6: e1001014.

9. Zou H (2006) The adaptive Lasso and its oracle properties. J Amer Stat Assoc 101: 1418-1429.

10. Neto EC, Ferrara CT, Attie AD, Yandell BS (2008) Inferring causal phenotype networks from segregating populations. Genetics 179: 1089-1100.

11. Shipley B (2002) Cause and Correlation in Biology: A User's Guide to Path Analysis, Structural Equations and Causal Inference. Cambridge University Press.

12. Spirtes P, Glymour C, Scheines R (2000) Causation, Prediction, and Search. Cambridge, MA: MIT Press, 2 edition.

13. Pickrell JK, Marioni JC, Pai AA, Degner JF, Engelhardt BE, et al. (2010) Understanding mechanisms underlying human gene expression variation with RNA sequencing. Nautre 464: 768-772.
