# Supplementary figures and images for: Inference of Gene Regulatory Networks with Sparse Structural Equation Models Exploiting Genetic Perturbations
Source: PLoS Comput Biol. 2013 May 23;9(5):e1003068. doi: 10.1371/journal.pcbi.1003068 (PMC3662697; doi:10.1371/journal.pcbi.1003068)

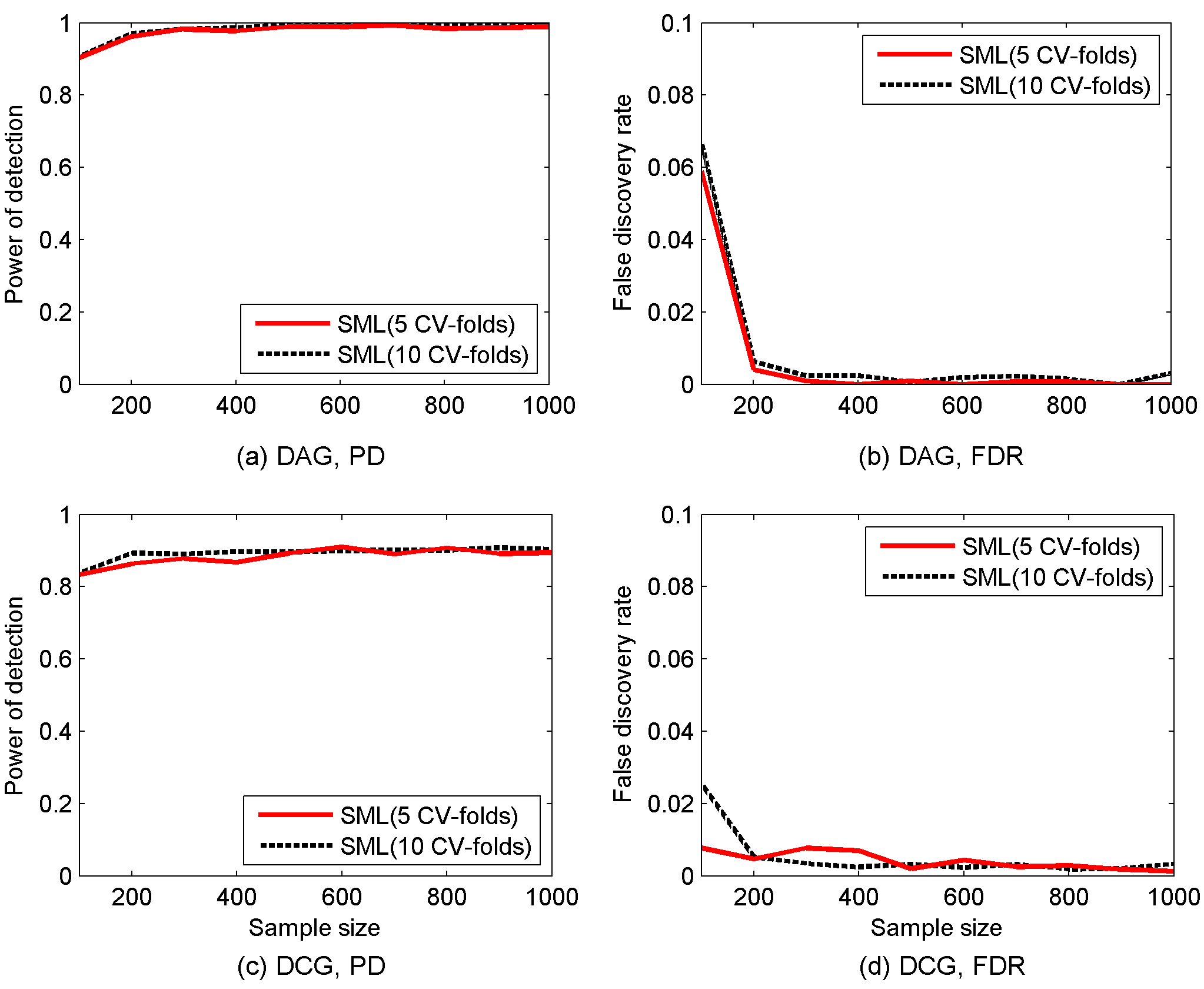

Supplement: Figure S1 — Performance of the SML algorithm for DAGs [(a) and (b)] or DCGs [(c) and (d)] of = 30 genes obtained with 5 (solid line) or 10 (dashed line) fold cross validation. Expected number of nodes per node is . PD and FDR were obtained from 100 replicates of the network with different sample sizes from 100 to 1,000. (TIF) [file pcbi.1003068.s002.tif]

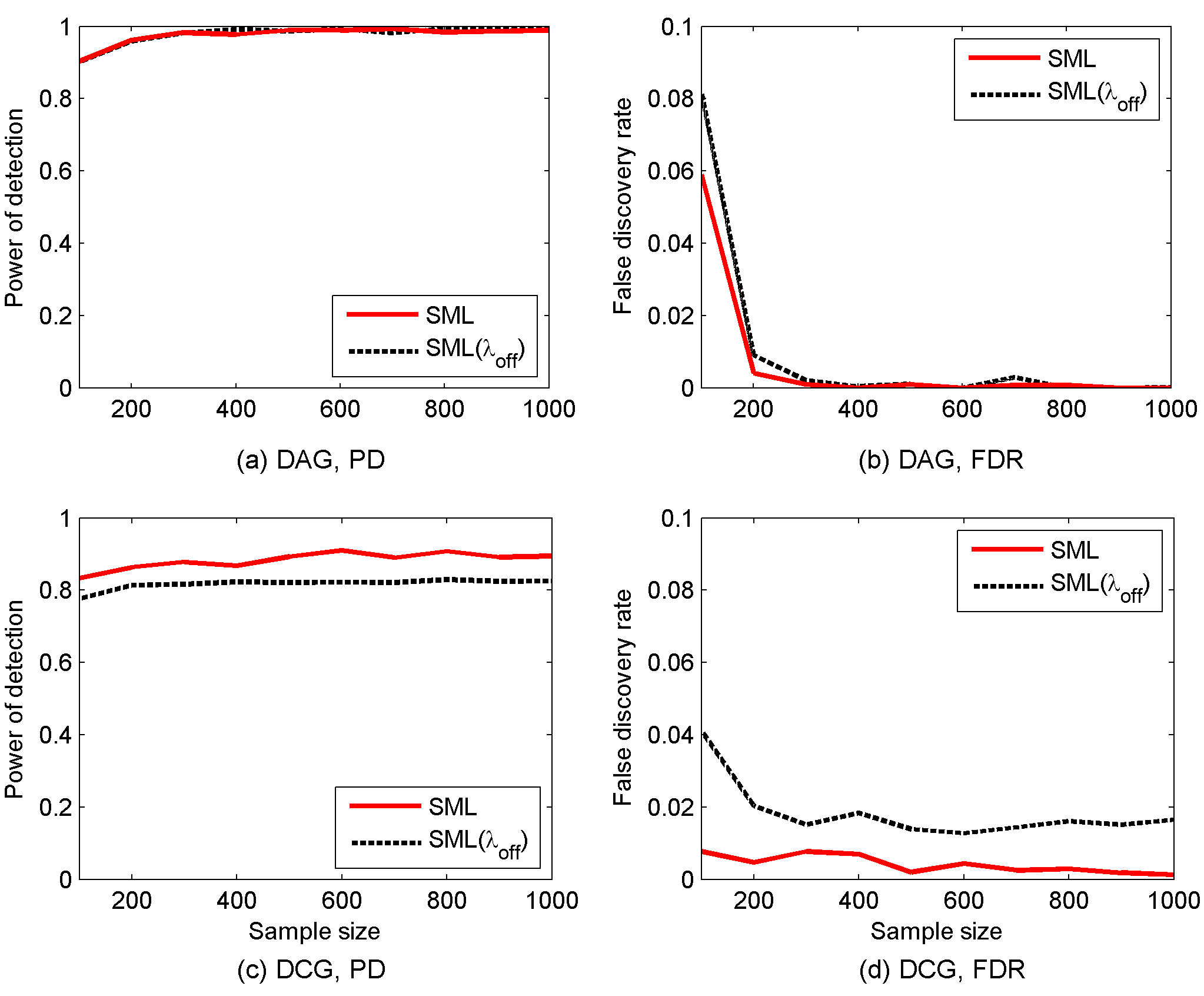

Supplement: Figure S2 — Performance of the SML algorithm for DAGs [ (a) and (b)] or DCGs [(c) and (d)] of = 30 genes obtained with the optimal (solid line) or an 10% less than the optimal (dashed line). Expected number of nodes per node is . PD and FDR were obtained from 100 replicates of the network with different sample sizes from 100 to 1,000. (TIF) [file pcbi.1003068.s003.tif]

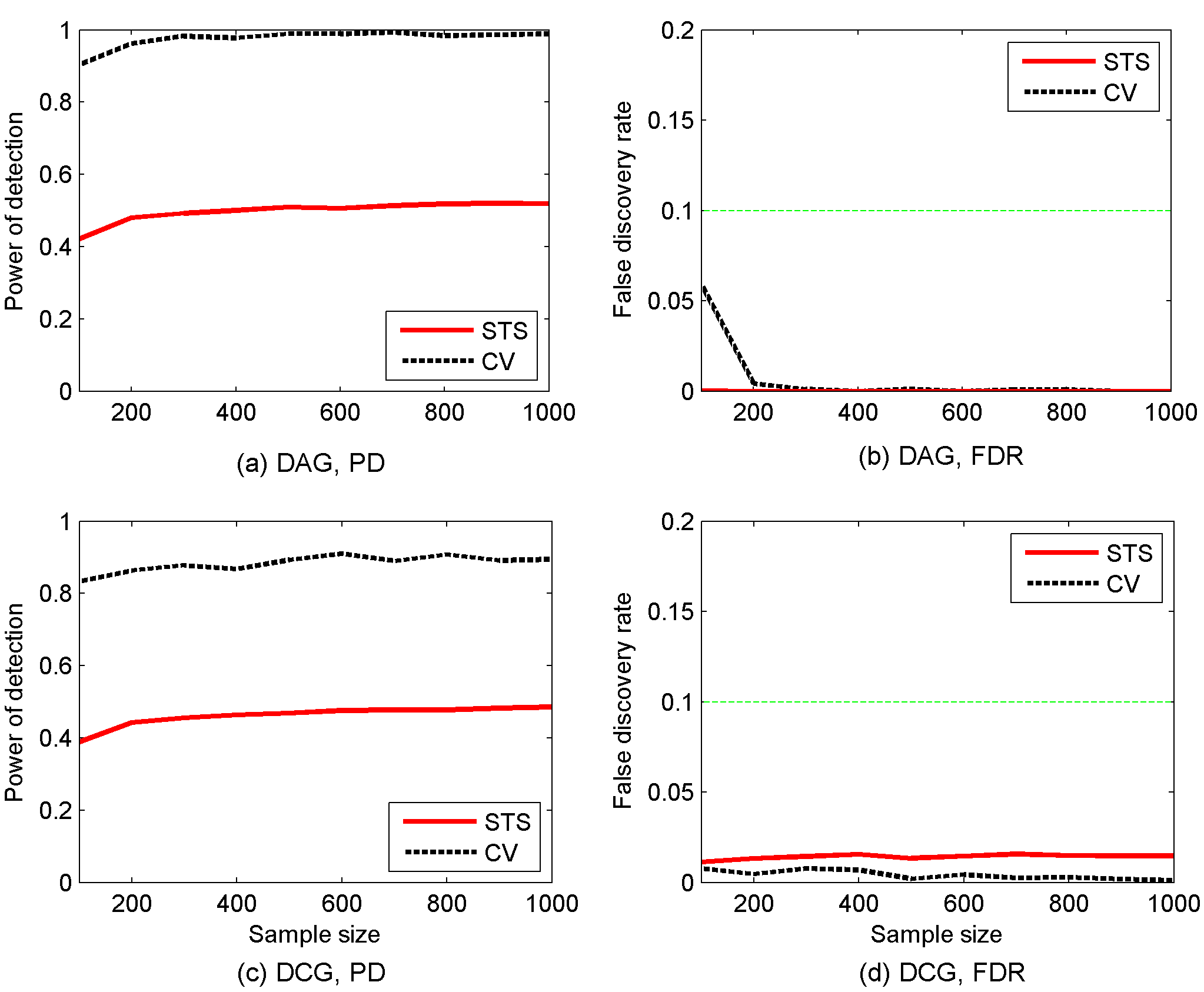

Supplement: Figure S3 — Performance of the SML algorithm with stability selection (STS) or cross validation for DAGs [ (a) and (b)] or DCGs [(c) and (d)] of genes. Expected number of nodes per node is . PD and FDR were obtained from 100 replicates of the network with different sample sizes from 100 to 1,000. (TIF) [file pcbi.1003068.s004.tif]

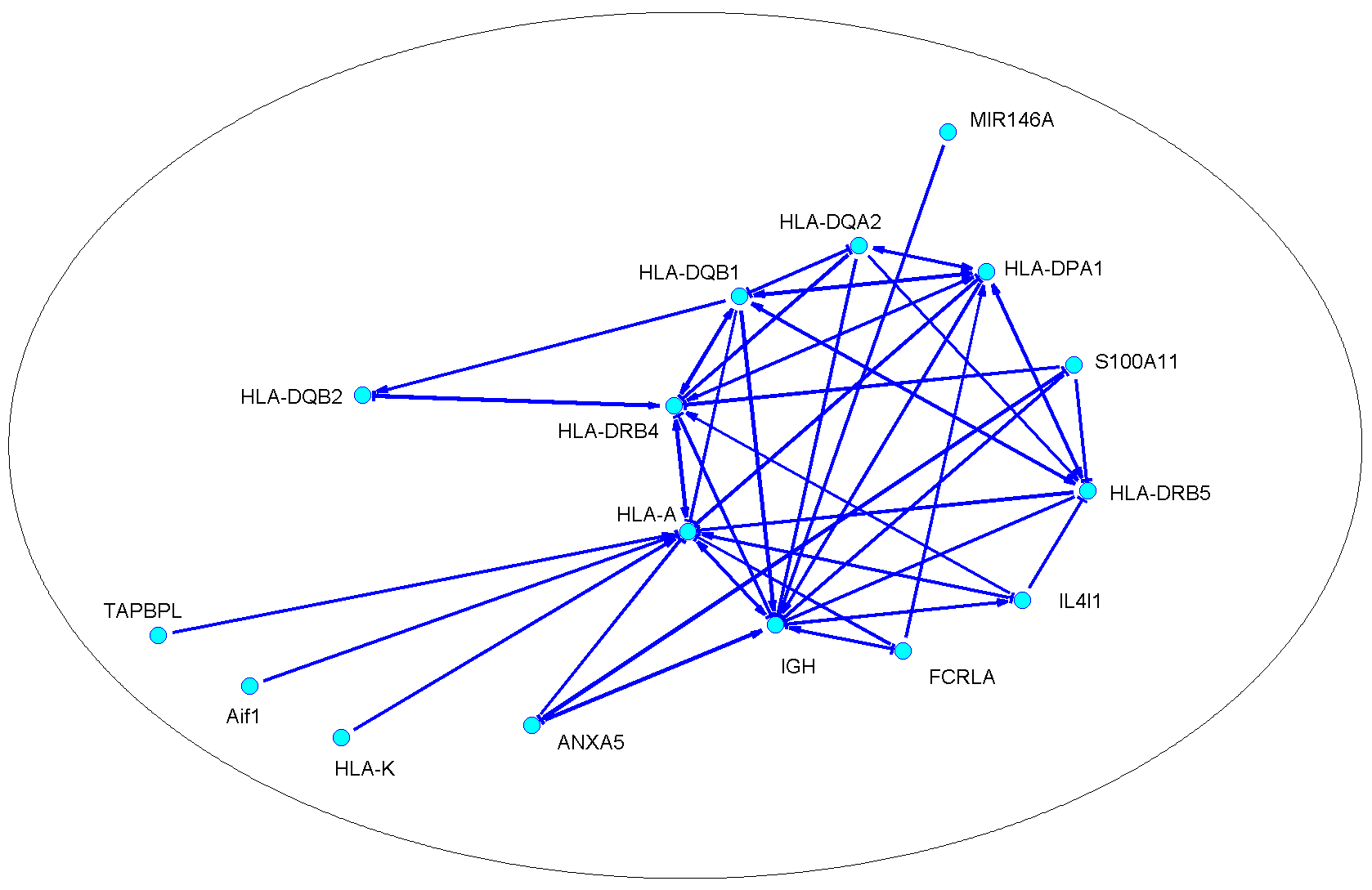

Supplement: Figure S4 — The network of 39 human genes inferred from gene expression and eQTL data with the SML algorithm. The 39 genes related to the immune system were chosen from [45] to have a reliable eQTL per gene. The SML algorithm was run with stability selection and edges were detected at an . See Table S1 for the IDs and description of 39 genes. IGH in this figure corresponds to gene ID ENSG00000211897. A edge stands for inhibitory effect and a edge stands for activating effect. (TIF) [file pcbi.1003068.s005.tif]
